# Contagious Caprine Pleuropneumonia in Endangered Tibetan Antelope, China, 2012

**DOI:** 10.3201/eid1912.130067

**Published:** 2013-12

**Authors:** Zhijun Yu, Tiecheng Wang, Heting Sun, Zhiping Xia, Kun Zhang, Dong Chu, Yu Xu, Yue Xin, Weiwei Xu, Kaihui Cheng, Xuexing Zheng, Geng Huang, Yongkun Zhao, Songtao Yang, Yuwei Gao, Xianzhu Xia

**Affiliations:** Military Veterinary Research Institute of Academy of Military Medical Sciences, Changchun, China (Z. Yu, T. Wang, Z. Xia, Y. Xin, W. Xu, K. Cheng, X. Zheng, G. Huang, Y. Zhao, S. Yang, Y. Gao, X. Xia);; Northeast Forestry University, Harbin, China (H. Sun);; Institute of Laboratory Animal Sciences of Chinese Academy of Medical Sciences & Peking Union Medical College, Beijing, China (Z. Yu, K. Zhang, X. Xia);; State Forestry Administration, Shenyang, China (D. Chu, Y. Xu)

**Keywords:** Tibetan antelope, Mycoplasma capricolum subsp. capripneumoniae, contagious caprine pleuropneumonia, bacteria, pneumonia, endangered, respiratory disease, CCPP, Mccp, China

**To the Editor:** Contagious caprine pleuropneumonia is a severe respiratory disease of goats caused by *Mycoplasma capricolum* subsp. *capripneumoniae* (Mccp), a member of the *M. mycoides* cluster (*1*). Mccp infection is associated with a 60% mortality rate and 90% illness rate, and the disease can cause substantial losses of livestock (*1*,*2*). We report a 2012 outbreak of contagious caprine pleuropneumonia in endangered Tibetan antelope (*Pantholops hodgsonii*) in China.

In 2000, the International Union of Conservation of Nature first listed the Tibetan antelope as an endangered species (*3*), and in 2004, the number of these antelope was estimated at 150,000 (*4*). Most Tibetan antelope live on China’s Qinghai–Tibet Plateau at an altitude of 3,700–5,500 m (*3*).

During September–December 2012, ≈2,400 endangered Tibetan antelope were found dead in the Naqu area of Tibet; the dead animals represented 16% of the 15,000 Tibetan antelope thought to live in the area. Necropsy was performed on 13 of the antelope at sites within the Shenzha, Shuanghu, and Nima localities of the Naqu area (Technical Appendix Table 1). Gross pathologic lesions were localized exclusively to the lung, where severe pleuropneumonia with partial hepatization was observed (Figure, panel A). The lungs of some affected antelope displayed a thickening of the interlobular septa, pleuritis, and an accumulation of straw-colored pleural fluid. The pleural exudate solidified to form a gelatinous covering on the lung (Figure, panel B).

**Figure:**
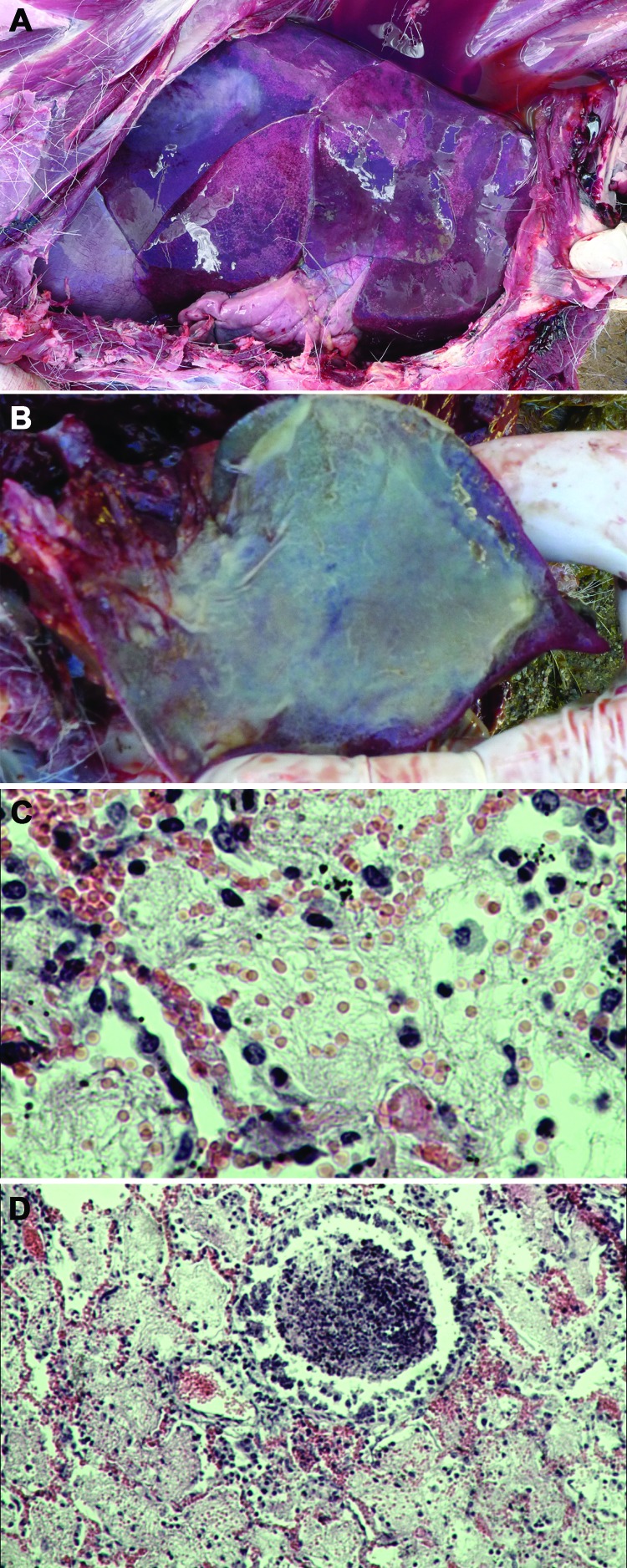
Pneumonia caused by *Mycoplasma capricolum* subsp. *capripneumoniae* in Tibetan antelope *(Pantholops hodgsonii*), Tibet, 2012. A) Lung of a caprine pleuropneumonia–infected Tibetan antelope (sample SZM2) showing lung hepatization. B) Lung of a caprine pleuropneumonia–infected Tibetan antelope (sample SH3) showing fibrin deposition. C and D) Fibrinous pneumonia with serofibrinous fluid and an inflammatory cell infiltrate, consisting of mainly lymphocytes, in the alveoli (panel C, sample SZM2, hematoxylin and eosin stain; original magnification ×400) and bronchioles (panel D, sample SH3, hematoxylin and eosin stain; original magnification ×100). Refer to Technical Appendix Table 1 for details of the lung samples used to generate images for this figure.

Samples of lung tissue from 5 of the antelope were selected for histologic examination. Four of the samples showed fibrinous pneumonia with serofibrinous fluid and an inflammatory cell infiltrate consisting mainly of lymphocytes in the alveoli (Figure, panel C) and bronchioles (Figure, panel D). One sample showed pulmonary edema with a protein-rich fluid effusion in alveoli.

Lung tissue from each of the 13 antelope was minced and inoculated into modified Hayflick broth, which has been used extensively to isolate *Mycoplasma* spp. from animals. Cultures were incubated at 37°C in a humidified atmosphere of 5% CO_2_ (*5*). The medium was examined daily by comparing inoculated broth with an uninoculated control broth. Moderate turbidity, a color change from pink to yellow, and an appreciable swirl of the culture when rotated were used as indicators of mycoplasma growth. After 2–3 passages in culture, 11 of 13 samples showed growth of mycoplasma. The presence of mycoplasma-like particles in the 11 growth-positive cultures was confirmed by electron microscopy (Technical Appendix Figure 1). Collectively, these observations implicated mycoplasma as the cause of disease in the affected antelope.

We next screened lung samples from each of the 13 Tibetan antelope by PCR for evidence of *M. mycoides* cluster and *M. bovis*. Eleven samples were positive for Mccp, but no other types of mycoplasma were detected (Technical Appendix Tables 1, 2). We conducted PCR as described (*6*) on the *arc*D gene of Mccp. In brief, we conducted 35 cycles of 30 s at 94°C, 15 s at 47°C, and 15 s at 72°C. Of note, lung sample SH7, which showed pulmonary edema, was negative for mycoplasma by PCR and culture. Lung samples from the 13 dead Tibetan antelope were also tested for an additional 16 potential pathogens (Technical Appendix Tables 1, 2) by PCR or reverse transcription PCR. No pathogens other than Mccp were detected.

To assess the relationship of the Mccp strain isolated from infected Tibetan antelope with previously isolated Mccp strains and the closely related *M. capricolum* subsp. *capricolum* (Mcc), we analyzed a 562-bp segment of the H2 gene of Mccp, which was used to distinguish the Mccp and Mcc as reported by Lorenzon et al. (*7*), isolated from an infected Tibetan antelope in Shuanghu county (sample SH3). The partial H2 sequence (GenBank accession no. KC441725) had higher sequence identity with Mccp isolates (99.3%–99.7%) than with Mcc isolates (90.2%–91.2% (Technical Appendix Figure 2). This phylogenetic analysis demonstrated that the Mccp isolated from infected Tibetan antelope belongs to the same clade as Mccp strains previously isolated in Africa and Asia.

The changing habitat of endangered Tibetan antelope may lead to increased exposure to Mccp, which can cause devastating outbreaks, such as the one reported here. Goats and sheep are herded on grasslands at an altitude of 4,300–5,000 m, the same area where Tibetan antelope reside. Goats are a reservoir for Mccp, and Mccp has been isolated from sheep in mixed herds with goats (*8*). Rail lines traverse the rangelands in this region, limiting the normal migration patterns of the Tibetan antelope population. Interaction among goats, sheep, and Tibetan antelope in this region, combined with the effect of human infringement on their rangeland, may increase the risk for disease emergence and transmission.

Our results show that contagious caprine pleuropneumonia may pose a substantial threat to the survival of endangered Tibetan antelope. Surveillance for Mccp infection among Tibetan antelope populations and domestic and wild goat and sheep populations that have close contact with the Tibetan antelope should be considered.

Technical Appendix Histopathologic findings and results of pathogen testing, primer pairs used for pathogen testing, lung tissue findings, and phylogenetic tree.
