# Supplementary material for: Contagious Caprine Pleuropneumonia in Endangered Tibetan Antelope, China, 2012
Source: Emerg Infect Dis. 2013 Dec;19(12):2051–3. doi: 10.3201/eid1912.130067 (PMC3840868; doi:10.3201/eid1912.130067)
Supplement: Technical Appendix — Histopathologic findings and results of pathogen testing, primer pairs used for pathogen testing, lung tissue findings, and phylogenetic tree. [file 13-0067-Techapp-s1.pdf]

Article DOI: http://dx.doi.org/10.3201/eid1912.130067

Contagious Caprine Pleuropneumonia in Endangered Tibetan Antelope, China

Technical Appendix

Technical Appendix Table 1. Histopathologic observations and pathogen testing of dead Tibetan antelopes evaluated during an outbreak of contagious caprine pleuropneumonia, China, 2012*

						PCR or reverse transcription PCR detection	
Sample	Locality*	GPS position	Sex, age, y	Histopathologic changes	Mccp isolated	Mccp	Additional pathogens†
SZM1	Shenzha	N30°54.777′, E08°21.170′	F, 5	Fibrinous pneumonia	+	+	–
SZM2	Shenzha	N30°54.776′, E08°21.167′	F, 4	Fibrinous pneumonia	+	+	–
SH1	Shuanghu	Not determined	F, 5	Not done	+	+	–
SH2	Shuanghu	N31°58.591′, E087°27.822′	M, 6	Not done	+	+	–
SH3	Shuanghu	N31°58.169′, E087°28.435′	M, 7	Fibrinous pneumonia	+	+	–
SH4	Shuanghu	N32°00.173′, E087°29.028′	M, 3	Fibrinous pneumonia	+	+	–
SH5	Shuanghu	N31°58.583′, E087°27.825′	F, 4	Not done	+	+	–
SH6	Shuanghu	N31°58.583′, E087°27.825′	F, 7	Not done	+	+	–
SH7	Shuanghu	Not determined	F, 9	Serous pneumonia	–	–	–
NM2	Nima	N31°58.256′, E087°22.439′	F, 3	Not done	+	+	–
NM3	Nima	Not determined	F, 6	Not done	+	+	–
NM4	Nima	N31°58.256′, E087°22.439′	F, 5	Not done	+	+	–
NM5	Nima	N31°57.492′, E087°22 164′	F, 4	Not done	–	–	–

*GPS, global position system; Mccp, *Mycoplasma capricolum* subsp. *Capripneumoniae*.

†Sixteen additional pathogens were tested by PCR or reverse transcription PCR: bluetongue virus, maedi-visna virus, goat arthritis encephalitis virus, foot and mouth disease virus, rinderpest virus, bovine parainfluenza virus 3, *Coxiella burnetii*, *Clostridium welchii*, *Pasteurella* spp., *Mycoplasma bovis*, *Mycoplasma leachii*, *Mycoplasma mycoides* subsp. *mycodies* large colony type, *Mycoplasma mycoides* subsp. *capri*, *Mycoplasma capricolum* subsp. *capricolum*, *Mycoplasma ovipneumoniae*, and *Mycoplasma mycoides* subsp. *mycoides* small colony type.

Technical Appendix Table 2. Primer pairs used for the detection of pathogens

Pathogen	Primers (5′→3′)
Bluetongue virus	BTV-251-F: TCGCTGCCATGCTATCCG
	BTV-251-R: CGTACGATGCGAATGCAG
Coxiella burnetii	IS111F1–485-F: TACTGGGTGTTGATATTGC
	IS111F1–485-R: CCGTTTCATCCGCGGTG
Maedi-visna virus	MVV-87-F: GAGGGATCAAGGATAAAAATGG
	MVV-87-R: GGTATCGYTGCAGYAACAT
Goat arthritis encephalitis virus	CAEV-296-F: CAAGCAGCAGGAGGAGAAGCTG
	CAEV-296-R:TCCTACCCCCATAATTTGATCCAC
Clostridium welchii	CPA-402-F: GTTGATAGCGCAGGACATGTTAAG
	CPA-402-R: CATGTAGTCATCTGTTCCAGCATC
Pasteurella spp.	C70/B37–1505-F: AGAGTTTGATYMTGGC
	C70/B37–1505-R: TACGGYTACCTTGTTACGA
Foot and mouth disease virus	FMD-ARS4-F: ACCAACCTCCTTGATGTGGCT
	FMD-NK61-R: GACATGTCCTCCTGCATCTG
Rinderpest virus	RPV-237-P1: ACAAACCNAGGATTGCTGAAATGAT
	RPV-237-P2: CTGAAYTTGTTCTGAAYTGAGTTCT
Bovine parainfluenza virus 3	RspV1/ MR2–739-F: GATCAGGAACTCTTAAAGGC
	RspV1/ MR2–739-R: TTTTCCCGACCCCTTCTAT
Mycoplasma bovis	Vsp-F: TGCTATTCATTTCTTTGTAGTATTTTATGT
	Vsp-R: TTTATTTCCTTTACCAATTACATATATTCG
M. leachii	7500bp1L: GTTGGTTTTGGATCAACTGG
	3480bp-R: TCTGATTTAGTTGGATTGAGTTCA
M. mycoides subsp. *mycodies* large colony type and *M.mycoides* subsp. *capri*	MMC2-L:CAATCCAGATCATAAAAAACCT<br>MMC1-R: CTCCTCATATTCCCCTAGAA
M. capricolum subsp. capricolum	MCCPL1-L: AGACCCAAATAAGCCATCCA
	MCCPL1-R: CTTTCACCGCTTGTTGAATG
M. mycoides subsp. *mycoides* small colony type	SC3NEST1-L: ACAAAAAGAAGATATGGTGTTGG
	SC3NEST1-R: ATCAGGTTTATCCATTGGTTGG
M. capricolum subspecies *capripneumoniae*	Mccp-spe-F: ATCATTTTTAATCCCTTCAAG
	Mccp-spe-R: TACTATGAGTAATTATAATATATGCAA
M. ovipneumoniae	LMFI: TGAACGGAATATGTTAGCTT
	LMRI: GACTTCATCCTGCACTCTGT
H2 gene partial of *M. capricolum subspecies capripneumoniae*	m-h2a:CGGGGATCCGGTATTGTTGTTGGAAGT
	m-h2b:CGGGTCGACGCTCCATCAAACATAGAT

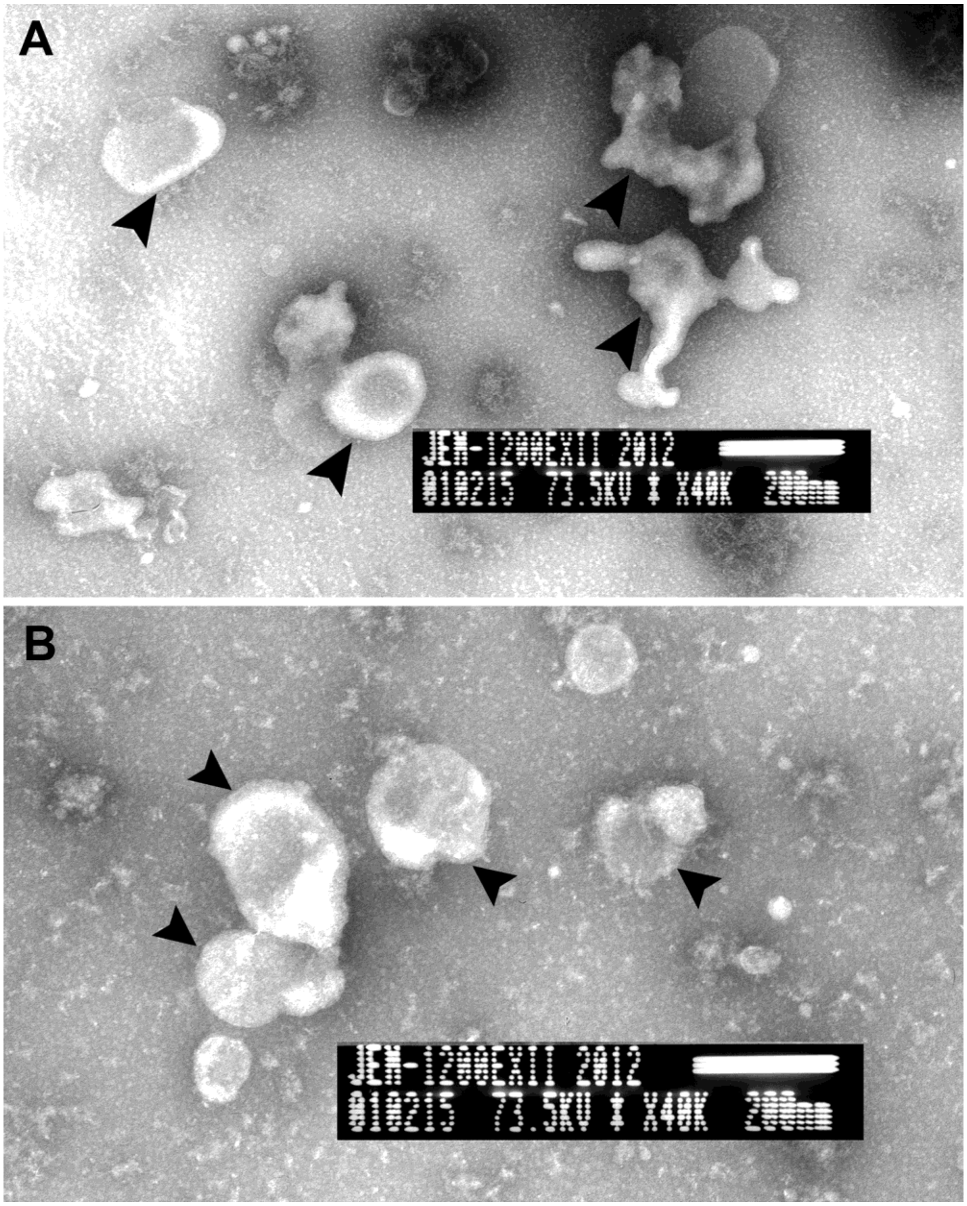

Technical Appendix Figure 1. Negative staining electron micrograph of *Mycoplasma capricolum* subsp. *capripneumoniae* particles. A) Electron micrograph of lung tissue of an infected Tibetan antelope SH3. Magnification ×40,000. B) Electron micrograph of culture supernatant after inoculation with tissue from the lungs of affected Tibetan antelope SH3. Magnification ×40,000. Arrowheads indicate pleomorphic mycoplasma-like particles appearing as short rods and spirals with diameters ranging from 100 to 300 nm. Scale bars indicate 200 nm. Refer to Technical Appendix Table 1 for details of the lung samples referred to in this Figure.

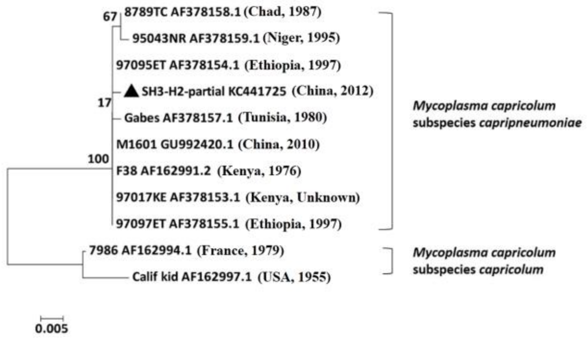

Technical Appendix Figure 2. Phylogenetic tree of *Mycoplasma capricolum* subsp. *capripneumoniae* (Mccp) and *M. capricolum* subspecies *capricolum* (Mcc) strains was generated by using partial H2 gene nucleotide sequences. Sequences are identified by strain name and GenBank accession number. The tree was created by using MEGA5 software (http://megasoftware.net/) according to the neighbor-joining method. Bootstrapping with 1,000 replicates was performed to determine the percentage reliability for each internal node. Horizontal branch lengths are proportional to genetic distances. The black triangle indicates the Mccp field isolate from an infected Tibetan antelope (sample SH3 in online Technical Appendix Table 1) in the Naqu area, China. Scale bar indicates nucleotide substitutions per site.
